# A lumbar puncture for all who are sleepy?

**DOI:** 10.1093/sleep/zsag162

**Published:** 2026-06-11

**Authors:** Mink S Schinkelshoek, Rolf Fronczek

**Affiliations:** Department of Neurology, Leiden University Medical Centre, Leiden, The Netherlands; Stichting Epilepsie Instellingen Nederland (SEIN), Sleep-Wake Centre, Heemstede, The Netherlands; Department of Neurology, Leiden University Medical Centre, Leiden, The Netherlands; Stichting Epilepsie Instellingen Nederland (SEIN), Sleep-Wake Centre, Heemstede, The Netherlands

In the diagnostic workup of Central Disorders of Hypersomnolence (CDH), we heavily rely on the Multiple Sleep Latency Test (MSLT). This test can be very useful in objectifying Excessive Daytime Sleepiness and detecting “pathological” intrusions of Rapid Eye Movement (REM)-sleep at the onset of a nap (Sleep-Onset-REM, SOREM). Because of this, the MSLT is historically treated as the practical diagnostic cornerstone for narcolepsy in many centers. However, we now know that assessing hypocretin deficiency in the cerebrospinal fluid (CSF) leads to a clear and homogenous diagnostic group: Narcolepsy Type 1 (NT1). This is the most classical form of narcolepsy previously called “Narcolepsy with Cataplexy.” However, to complicate matters, not all people with hypocretin deficiency (NT1) have recognized cataplexy.

In most sleep centers, CSF is not routinely assessed in the diagnostic workup. This is often due to logistical reasons (i.e. it is not possible/feasible) but can also be because clinicians deem the clinical history and the MSLT results sufficient. Biscarini and colleagues challenge this idea [1]. They describe 10 years of diagnostic experience of the Bologna Narcolepsy Center. This consists of 870 cases undergoing a standardized work-up for CDH including CSF assessment. Of these, 464 had an MSLT “negative” for narcolepsy. Still, hypocretin deficiency was found in 34 of those. This led to reclassification of 7 per cent of cases. Interestingly, these cases more often lacked the HLA type typical for NT1, had fewer SOREMs and higher hypocretin-1 CSF levels; although still below the 110 pg/mL cut-off. Importantly, 15 per cent of these cases did not have cataplexy. Especially these MSLT-negative cases without cataplexy would have been missed if CSF assessment had not been performed.

## The MSLT: Useful, but Not Definitive

The past two decades, research groups have questioned whether MSLT findings adequately capture the biological substrate of narcolepsy [2–4]. It was demonstrated that non-specific factors, such as insufficient sleep, psychiatric disease, circadian phase misalignment, age, sleep fragmentation and medication washout can influence MSLT performance significantly [5–8]. In parallel, reproducibility of the test proved worse than expected, with test–retest studies consistently only showing relatively stable results in patients with cataplexy and hypocretin deficiency. In NT2 and idiopathic hypersomnia, in contrast, diagnostic conversion when repeating MSLT was common. Longitudinal cohorts further demonstrated that part of the patient population initially classified as NT2 subsequently meet criteria for NT1, most commonly by the onset of typical cataplexy, raising concerns that current MSLT-defined categories insufficiently capture the underlying pathophysiology [9, 10].

This is further supported by multiple studies comparing MSLT findings with CSF hypocretin-1 levels in which levels <110 pg/mL—and thus a definite NT1 diagnosis—may occur despite negative MSLT findings. Others demonstrated that SOREMP burden, the presence or absence of (typical) cataplexy, and HLA-DQB1*06:02 status predict hypocretin deficiency more accurately than sleep latency alone [8, 11–13]. Recently, it was demonstrated that spontaneous SOREMPs recorded during prolonged daytime or 24-h polysomnography may identify narcolepsy cases missed by conventional MSLT protocols [12, 14]. Together, the literature increasingly supports the view that the MSLT, while clinically useful, represents an imperfect proxy for a biologically heterogeneous disease spectrum rather than a definitive diagnostic gold standard.

## Hypocretin As Biomarker: Closer to Biology, but Not Without Caveats

Although the MSLT is standardized and non-invasive, its limitations have become increasingly clear. At the same time, the field has not yet identified a universally applicable non-invasive alternative. Even though it best reflects the biological process underlying the disease, CSF hypocretin measurement is invasive, technically challenging, and methodologically imperfect; studies using mass spectrometry and chromatographic approaches have shown that conventional radioimmunoassays quantify multiple hypocretin-derived peptides rather than the intact hypocretin-1 alone, complicating interpretation of intermediate values and questioning the validity of diagnostic thresholds [15–17]. The existence of patients with cataplexy but preserved hypocretin values either underscores the imperfection of the measurement of hypocretin itself or suggests that NT1 pathophysiology may not be as uniform as we now assume.

## Hypocretin Receptor 2 Agonists

Yet, considering the upcoming hypocretin receptor 2 agonists, it may become increasingly important to know which CDH patients are actually hypocretin deficient. The expected effects and correct dosages of this new drug class may very well depend on this. The work from Biscarini and colleagues therefore suggests that CSF assessment should perhaps be performed more often. In the current clinical reality, CSF hypocretin-1 levels would already be assessed in most sleep centers in case of a suspicion of cataplexy despite a negative MSLT. However, the work from Bologna shows that in about 1.5 per cent of cases hypocretin deficiency might still be missed when solely relying on the MSLT and the presence of cataplexy. The question remains whether this should urge all sleep centers to perform lumbar punctures as part of the routine CDH work-up.

Biscarini and colleagues do not necessarily make the case for lumbar puncture in every sleepy patient. They do, however, make a strong case against diagnostic complacency after a negative MSLT. In patients with persistent clinical suspicion of narcolepsy, especially when cataplexy is subtle or absent, HLA-DQB1*06:02 is present, or any SOREMP has been recorded, CSF hypocretin measurement remains the closest available route to biological certainty. Until better and robust non-invasive biomarkers emerge, the question is not whether all sleepy patients need a lumbar puncture, but which MSLT-negative patients we can no longer afford not to test.

## Disclosure statement

*Financial disclosure*: RF did consultancy work for Jazz Pharma & Takeda, and participated in a safety review committee for Centessa. No funding was obtained for this editorial.

*Non-financial disclosure*: The authors report no non-financial potential conflicts of interest.
